# Left Ventricular Twist and the “Rigid Body Rotation” Pattern in Patients Treated with Anthracyclines or Anti-HER2

**DOI:** 10.3390/jcm13113352

**Published:** 2024-06-06

**Authors:** Federico Guerra, Giulia Stronati, Alice Frangione, Edlira Rrapaj, Marco Flori, Michele Alfieri, Samuele Principi, Alessandro Barbarossa, Giuseppe Ciliberti, Antonio Dello Russo

**Affiliations:** 1 Cardiology and Arrhythmology Clinic, Marche University Hospital, 60126 Ancona, Italy; guerra.fede@gmail.com (F.G.); alice.frangione@ospedaliriuniti.marche.it (A.F.); edlira.rrapaj@ospedaliriuniti.marche.it (E.R.); alessandro.barbarossa@ospedaliriuniti.marche.it (A.B.); antonio.dellorusso@gmail.com (A.D.R.); 2 Department of Biomedical Sciences and Public Health, Marche Polytechnic University, 60121 Ancona, Italy; giulia.emily.stronati@gmail.com (G.S.); m.alfieri95@gmail.com (M.A.); 3 Cardiology Unit, “Santa Maria della Misericordia” Hospital, 61029 Urbino, Italy; marco.flori@sanita.marche.it

**Keywords:** anthracycline, cardiotoxicity, chemotherapy, heart failure, speckle tracking, twist

## Abstract

**Background:** During the physiological cardiac cycle, the helix orientation of the muscle fibres induces the rotation of the apex relative to the base of the left ventricular (LV). In heart failure, LV torsion is impaired, and rotation at basal and apical levels occurs in the same direction, a phenomenon called rigid body rotation (RBR). We aimed to evaluate whether the RBR pattern and GLS together could improve the diagnosis of cardiotoxicity in patients treated with anthracyclines and/or anti-HER2. **Methods:** With an observational, retrospective study involving 175 patients (mean age 55 ± 12 years, 94% females), we evaluated the development of cancer therapeutic–related cardiac dysfunction (CTRCD) defined according to ESC guidelines. We characterised LV dysfunction by echocardiographic standard and speckle-tracking (GLS and RBR pattern) measurements. Patients with a previous diagnosis of structural heart disease or atrial fibrillation were excluded. **Results:** At the time of enrolment, the chemotherapy regimen included trastuzumab (96%), pertuzumab (21%), and anthracyclines (13%). Twenty-two patients (12.5%) developed cardiotoxicity, and thirteen patients developed an RBR within 6 months of follow-up. In all cases, the RBR pattern was associated with cardiotoxicity (*p* < 0.001), reporting an optimal specificity but poor sensitivity at three and six months. However, the addition of the RBR pattern to the global longitudinal strain (GLS) ≥ −16% increased the odds ratio (OR) from 25.6 to 32.6 at three months and from 32.5 to 49.6 at six months rather than GLS alone. **Conclusions:** The RBR pattern improves the diagnostic accuracy of GLS for the detection of cardiotoxicity secondary to anthracyclines and anti-HER2-based treatments.

## 1. Introduction

Cardiovascular disease and cancer are the most prevalent diseases in the developed world, and together, they are the cause of two-thirds of deaths worldwide [1]. These conditions are interlinked through common risk factors, often coincident in ageing populations. Multiple cardiovascular comorbidities can profoundly influence cancer care management, and many anticancer treatments can substantially affect the heart and vascular system [1,2]. Of course, before beginning chemotherapy treatment, a complete medical history and physical examination should be obtained to appropriately risk-stratify patients, balance any potentially adverse cardiovascular effects against the benefits, and establish the best cardiac surveillance [1,3]. Myocardial dysfunction and heart failure (HF) are the most feared complications with the heaviest prognostic impact. HF because of cancer therapy has been linked to a 3.5-fold increase in mortality risk compared with idiopathic cardiomyopathy [4,5]. Some effects could be limited to exposure to the drug, while others could appear later [6]. In this setting, echocardiography is the cornerstone of the diagnostic process. Unfortunately, wide consensus regarding the best parameter to detect cardiotoxicity has still to be reached, as left ventricular ejection fraction (LVEF) has two important limitations [6,7,8]. First, it has a non-negligible inter- and intra-observer variability. Second, LVEF reduction is often a late phenomenon of cardiotoxicity. For those reasons, many studies have evaluated bi-dimensional speckle-tracking analysis to describe left ventricle (LV) deformation. This technique offers several parameters for a better characterisation of cardiac function. GLS was found to be useful in the early diagnosis of cancer therapy-related cardiac dysfunction (CTRCD) in anthracycline- and anti-HER2-treated patients [6]. The analysis of the LV torsion still has an indeterminate meaning in this field. In normal hearts, there is a clockwise rotation of the LV base and a counterclockwise rotation of the LV apex during systole [9]. LV twisting measures the maximum instantaneous difference between the base and apex rotation and could potentially be useful in the early detection of cardiac dysfunction [10]. When LV torsion is impaired, an RBR pattern can be detected. In these patients, there is a loss of twisting and rotation at basal and apical levels that occurs in the same clockwise or counterclockwise direction [11]. The aim of the present study was to evaluate whether the RBR pattern and GLS together could improve the diagnosis of cardiotoxicity in patients treated with anthracyclines and/or anti-HER2.

## 2. Materials and Methods

### 2.1. Population and Enrolment

This was an observational, retrospective, single-centre study. We selected patients from among those referred to the cardio-oncologic outpatient unit of our institute from October 2014 to May 2020. Inclusion criteria were age ≥ 18 years old, no previous diagnosis of cardiomyopathy or structural heart disease, and scheduled neo-adjuvant or adjuvant chemotherapy with anthracyclines and/or anti-HER2. Patients with atrial fibrillation and who had suboptimal echo acquisitions were excluded from the study to perform accurate bi-dimensional speckle-tracking measurements.

### 2.2. Study Protocol

The study protocol consisted of three complete cardiologic evaluations in 12 ± 3 months: The first one was performed before the start of cancer treatment, or when this was not possible, we chose the assessment closest to the beginning of the chemotherapy. The next follow-up visit was performed 3 months later and the last one 12 months after the start of the drugs. The cardiologic evaluation included medical history collected with particular attention to cardiovascular risk factors and comorbidity, physical examination, blood pressure measurement, 12-lead ECG, collection of biomarkers, and a complete echocardiography exam. The biomarkers of interest to us were troponin I, CK-MB, and creatinine from which we calculated the glomerular filtration rate using the MDRD formula. In the case of CTRCD, we applied an internal protocol resulting from the experience of our cardio-oncology unit and the review of the most recent scientific evidence [1,2,12]. Chemotherapy was stopped in all patients developing cardiotoxicity with an LVEF < 40%, and in patients treated with anthracyclines and with LVEF between 40 and 49% or GLS > −16%, we stopped just the anthracycline-based treatment. All patients with CTRCD started cardioprotective treatments, and all patients with LVEF < 40% were treated according to the ESC recommendations.

### 2.3. Echocardiography

The ultrasound instruments were a Vivid 7 Pro (GE Medical Systems, Milwaukee, WI, USA) with a 4 MHz monoplane transducer (M4S) or a Vivid E80 (GE Medical Systems, Milwaukee, WI, USA) provided with a 5 MHz monoplane transducer (M5S). The echocardiographic images were obtained with the patient supine and in left lateral decubitus at a frame rate of 40–90 frames per second at the end of a normal breath. Trained cardiologists collected the images of at least three consecutive beats. A single operator collected echocardiographic standard measurements and evaluated them according to the most recent recommendations. The same operator performed the speckle-tracking analysis offline with adequate software (EchoPAC BT201; GE Medical Systems, Milwaukee, WI, USA) according to the manufacturer’s protocol.

### 2.4. Standard Echocardiography

In the parasternal long-axis view, internal measurements of the left ventricle and its walls were taken using the M-mode approach during both the systole and the diastole. The LV mass was obtained with the Cube formula. The right function was assessed with the TAPSE. LVEF was calculated from the average of a minimum of three complete sets of measurements obtained by the Simpson biplane method, and then the obtained volumes were indexed for the BSA. Right and left atrial areas were measured in four chambers. Systolic pulmonary artery pressure was estimated through the maximum velocity of the trans-tricuspid regurgitant flow and the mean right atria pressure. The diastolic function was evaluated according to the “Recommendations for the Evaluation of the Left Ventricle Diastolic Function of Echocardiography”, and the values of E/A and E/e′ were calculated [13]. Limited to the ejection fraction (and the start of the protocol before any ESC guidelines), we considered CTRCD as a decrease of >10 percentage points to a value below the absolute value of 50% [7].

### 2.5. Two-Dimensional Speckle-Tracking Echocardiography and Torsion Analysis

Speckle-tracking analysis software was used to assess GLS and LV torsional movements. GLS was derived from three standard apical views: 4 chambers, 2 chambers, and the apical long axis. For the measurement of LV twisting, two parasternal short-axis images were recorded: one at the basal level (most circular cross section showing the tips of the mitral valve leaflets) and the other at the apical level (image without visible papillary muscles just proximal to the level where the LV cavity disappears at the end-systolic period). The basal and apical images were selected to match similar RR intervals. Speckle-tracking analysis was performed by manually tracing the LV endocardial border. If at least one segment was considered inadequate by the software, manual improvement of endocardial tracking was attempted a second time. A maximum of one segment could be rejected for every single exam.

**Figure 1:**
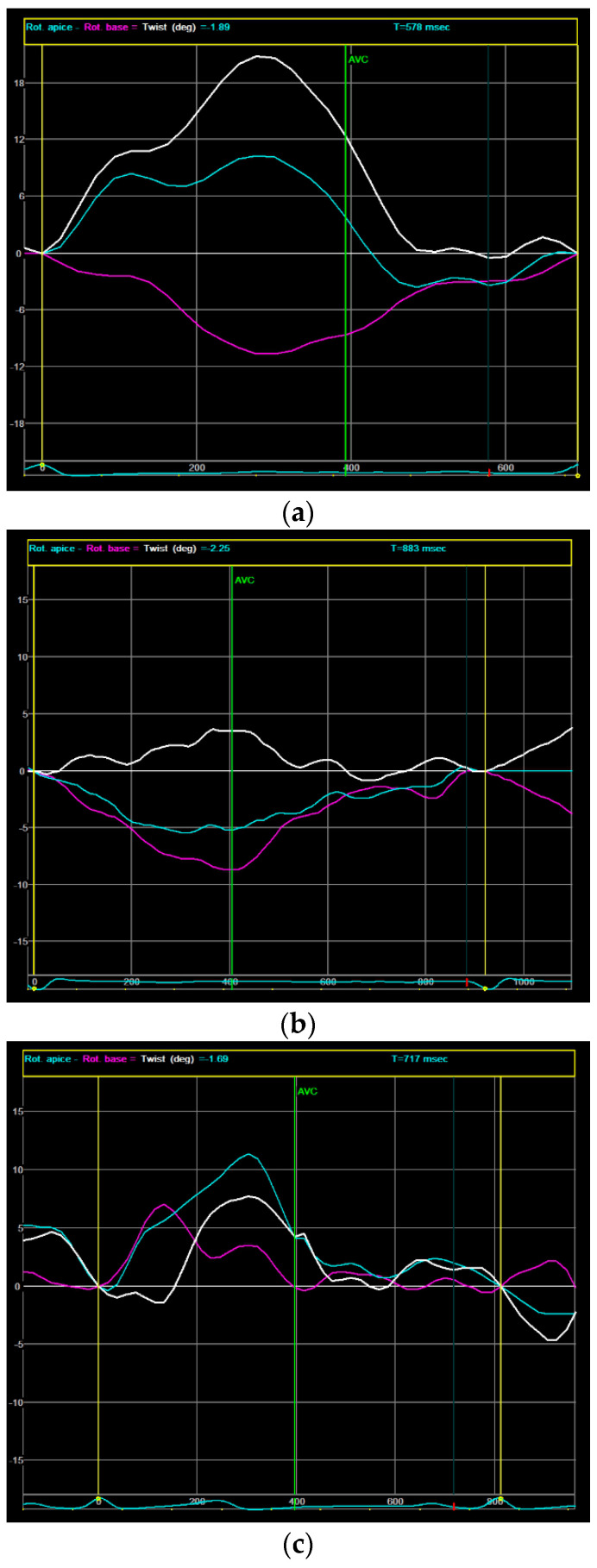
LV torsion speckle-tracking analysis: (**a**) a normal pattern with a clockwise rotation of the LV at the basal level (purple line) and a counterclockwise rotation of the apex (light blue line); (**b**) an RBR pattern due to reversed (clockwise) apical rotation; (**c**) an RBR pattern due to reversed (counterclockwise) basal rotation. The purple line (base) and the blue line (apex) represent the degree of rotation in a clockwise (below the zero line) or in a counterclockwise (above the zero line) direction. The white line represents the total twist generated by the left ventricle as the difference between the apical and basal rotations.

For every echocardiographic study, we calculated the GLS, and the peak systolic LV twisting was calculated as the maximum instantaneous difference between peak systolic apical and basal rotation. An RBR pattern was defined as the loss of opposite rotation between the LV base and LV apex [11]. Figure 1 shows three speckle-tracking analyses of the LV twisting: a normal pattern (Figure 1a), an RBR pattern due to reversed apical rotation (Figure 1b), and an RBR pattern due to reversed basal rotation (Figure 1c).

### 2.6. Statistical Analysis

Quantitative variables were checked for normality by the Kolmogorov–Smirnov test and described as mean ± standard deviation or median and interquartile range, as appropriate. A general linear model for repeated measures was used to assess time-dependent changes during follow-up. Categorical variables were assessed by using χ^2^ analysis and described as prevalence in the total population. Binary logistic regression analysis was used to create adjusted models including independent variables associated with cardiotoxicity. The receiver operating characteristic (ROC) curve was used to define the sensitivity and specificity of various cut-off values for both longitudinal and torsion strain parameters. Values of *p* < 0.05 (two-tailed) were considered statistically significant. SPSS 25.0 for Windows (SPSS Inc., Chicago, IL, USA) was used for statistical analysis.

## 3. Results

### 3.1. Population and Pharmacological Treatment

A total of 175 patients met the inclusion and exclusion criteria described previously. There were just 11 men, and the mean age of the population was 55 years old. Of the 175 individuals, 159 had breast cancer (154 with an invasive ductal type, 4 with invasive lobular carcinoma, and 1 with mixed breast cancer), 12 had melanoma, 2 had non-Hodgkin’s lymphoma, 1 had acute myeloid leukaemia, and 1 had hepatocellular carcinoma. In the patients’ chemotherapy protocols at the time of enrolment, the prevalence of drugs was 13% for anthracycline, 96% for trastuzumab, 21% for pertuzumab, 58% for taxans, 13% for cyclophosphamide, 8% for 5-fluorouracil, and 4% for vincristine. Of the 152 patients who were not on anthracyclines at the time of enrolment, 143 (94%) had previously received a chemotherapy-based regimen as a first-line therapy.

The main demographical and clinical characteristics of the population subdivided based on a subsequent development of cardiotoxicity, or lack thereof, are shown in Table 1.

At each visit, patients underwent a complete echocardiographic examination. The main parameters obtained from the echocardiogram performed at the baseline assessment were examined in the statistical analysis. These include LVEF; the end-diastolic and systolic volume of the left ventricle; the area of the right atrium and the left atrium; Doppler parameters for the study of the transmitral pattern and the filling pressure (E wave, A wave, E/A ratio, and E/E′ ratio); the function of the right ventricle (TAPSE); systolic pulmonary arterial pressure (PAP); and the parameters of myocardial deformation, namely GLS, stated as the median value (GLS AVG) and LV rotation parameters and twisting. The basal and apical rotation of the left ventricle in systole and diastole were expressed in degrees (°). The data relating to these measurements, reported as an average value and divided according to the future presence or absence of cardiotoxicity, are shown in Table 2.

The statistical analysis carried out on the parameters described above revealed no statistically significant differences at baseline among those patients who developed cardiotoxicity and those who did not develop it (*p* > 0.05).

### 3.2. Cardiotoxicity

**Figure 2:**
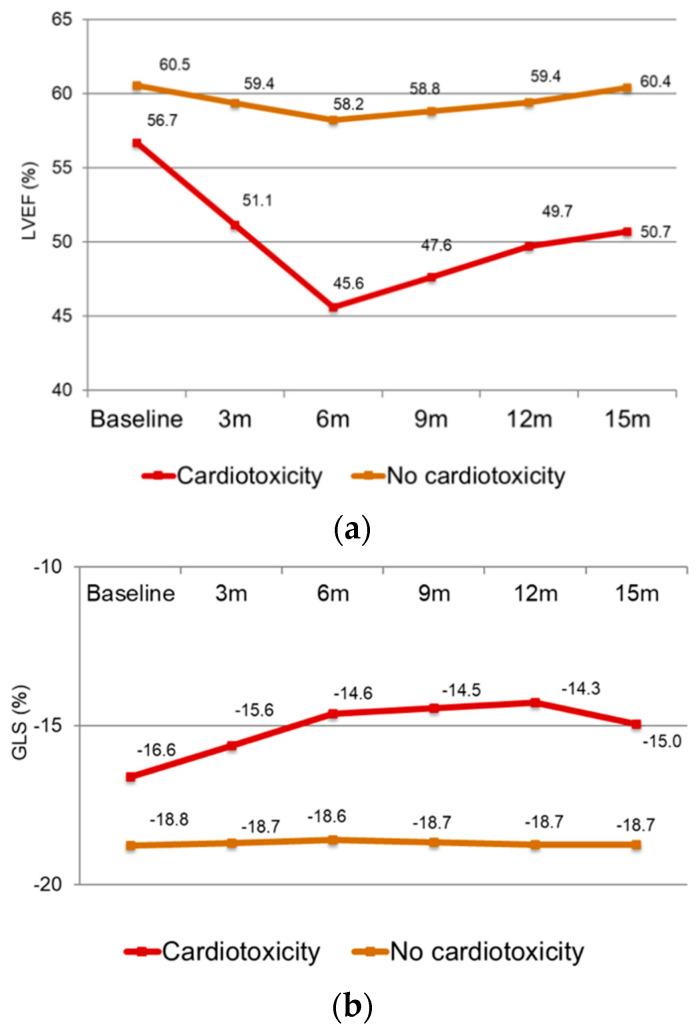
LV ejection fraction (**a**) and GLS (**b**) over the follow-up, according to the development of CTRCD.

At the end of the follow-up (median 15 months, 1st–3rd quartile, 12–21 months), 22 patients out of the 175 patients (12.7%) developed CTRCD [7]. All patients with CTRCD started cardioprotective treatments, and chemotherapy was stopped according to the protocol previously described. The average values of LVEF at enrolment and subsequently every 3 months up to the 15th month were calculated and compared in the two groups of patients, based on the development of CTRCD (Figure 2a). The same type of analysis was performed for the evaluation of the average GLS trend, compared in the two groups (Figure 2b). For every echocardiogram, we analysed LV torsion looking for the RBR pattern, and we found it in 13 patients: 7 people at 3 months and 6 people at 6 months.

The GLS and rotation mechanics (RBR) were then analysed in an isolated and combined manner to evaluate their diagnostic power in predicting anthracycline and HER2-induced CTRCD. Table 3 shows the results in terms of odds ratio (OR), sensitivity (SE), specificity (SP), and predictive positive value and negative value (PPV and PNV). ROC curve analysis confirmed GLS as a good predictive factor for further development of CTRCD: A cut-off of −16% or higher showed a sensitivity of 73% and a specificity of 90% at 3 months with an OR of 25.6 and a sensitivity of 77% and a specificity of 91% at 6 months with an OR of 32.5. RBR development was also associated with CTRCD (*p* < 0.01) at 3 and 6 months after the beginning of the follow-up. The RBR pattern showed optimal specificity both times but with a low sensitivity. However, when the presence of either RBR pattern and GLS ≥ −16% were used together as a combined criterion, it was possible to predict CTRCD with good sensitivity: 77% and 86% at 3 and 6 months, respectively. The OR of the two combined parameters increased significantly, reaching values of 32.6 at 3 months and 49.6 at 6 months.

## 4. Discussion

Modern oncologic treatments have improved cancer-related survival, but their clinical effectiveness may be thwarted by the development of cardiotoxicity that negatively affects patients’ outcomes [9,10,11,12,13]. Cardiac damage may not become apparent until years or even decades after receiving the cardiotoxic treatment. This is shown to be particularly true in adult survivors of childhood cancers [6]. In the diagnostic process of CTRCD, echocardiography is the cornerstone strategy for monitoring cardiac function because of its wide availability, easy repeatability, versatility, lack of radiation exposure, and safety in patients with accompanying renal disease [6]. Despite this, some limitations remain, such as the absence of a unique definition of echocardiographic cardiotoxicity [1,2,6] and the fact that LVEF often fails to detect small changes in LV contractility. It is also important to bear in mind the preload dependency of this measurement. Moreover, the heart has significant cardiac reserve, and the expression of damage in the form of alterations in systolic or diastolic parameters may not be apparent until a substantial amount of cardiac reserve has been exhausted [14,15]. This is why LVEF reduction is often a delayed phenomenon of cardiotoxicity [6].

On the other hand, early cardiotoxicity detection and its prompt treatment seem to be crucial for a major improvement in cardiac function [11]. Guerra et al. showed that GLS ≥ −16 at 3 months was able to predict which patient would have a significant LVEF reduction at 6 or 9 months [16]. According to Thavendiranathan et al., the thresholds of change in GLS to predict cardiotoxicity range from 10% to 15% using speckle tracking [8].

In the present paper, we investigated the added value of rotational mechanics to GLS in predicting chemotherapy-induced cardiotoxicity. In a normal contracting LV, the base and apex rotate in opposite directions during ventricular ejection: clockwise for the base and counterclockwise for the apex.

This torsional motion of the whole LV is due to the helical architecture of the LV myocardium: right-handed fibres in the endocardium and left-hand fibres in the epicardium. The direction of the twist depends on the prevalence of the epicardial muscle fibres [17]. Basal and apical LV rotations are expressed in degrees, as is twisting, which is their algebraic sum. Torsion is obtained by dividing twisting by the length of the LV cavity in degrees per centimetres [9]. Hirohiko M. et al. have already suggested that LV torsion analysis could be a useful non-invasive approach for the early detection of subclinical anthracycline cardiotoxicity [18].

LV rotation gradually changes from clockwise at the mitral level to counterclockwise at the apex, and therefore the level of sampling is critical in determining the magnitude of rotation. In particular, the apex seems to account for the greatest amount of rotation and may be the most important source of error [19]. Parisi et al. reported low reproducibility of torsion when comparing bi- and tri-dimensional acquisitions [20]. Therefore, a reproducibility issue must be considered, limiting the use of absolute values of rotational parameters in clinical practice [10]. In our analysis, we tested a simpler parameter that is nonetheless the expression of complete failure of rotational mechanics: the RBR or solid body rotation pattern defined as the loss of the opposite rotation of the base and apex. It is less dependent on absolute measurements. Bas M. et al. found a complete concordance between different observers in determining the direction of apical rotation [21]. The RBR phenomenon has been described in myocardial non-compaction [21] and heart failure in cardiac resynchronisation therapy candidates [22]. We found that the RBR pattern had low sensitivity but very high specificity as a marker of systolic dysfunction in patients treated with anthracyclines and/or trastuzumab. Its presence is strongly suggestive of CTRCD while the absence thereof does not exclude the development of the left ventricular. Moreover, this parameter seems GLS-independent in predicting cardiotoxicity (*p* < 0.05). These two characteristics define its additive value to GLS analysis: The OR associated with GLS increased from 25.6 to values of 32.6 with RBR at 3 months and from 32.5 to 49.6 at 6 months. This combination confirms Mornons’ intuition. He proposed the combined index GLS×LVtw to predict anthracycline cardiac toxicity [21]. In our study, we also observed that basal GLS was about −16% in those patients who would go on to develop left ventricular dysfunction, whereas it was about −18% in those who would not. This is in line with the cut-off GLS ≥ −16% proposed by Guerra et al. as an indicator of LV dysfunction. In the case of CTRCD, GLS showed a significant early decrease with a slight recovery after 15 months. This underlines the consistent and persistent alteration of GLS. With this study, as Table 1 and Table 2 show, we did not find any basal clinical characteristic associated with the development of CTRCD.

### Limitations

We should underline some limitations: This is a single-centre and retrospective study with a limited number of patients. Therefore, a power calculation was deemed unfeasible, and some secondary analyses were not performed due to the low sample size of the subgroup with cardiotoxicity. Secondly, other cardiovascular complications such as arterial hypertension and arrhythmias could have influenced both systolic dysfunction and strain deformation parameters. Thirdly, like all ultrasound-based analyses, the RBR pattern is operator-dependent, and it relies on good image acquisition [10]. Moreover, while previous research reported good reproducibility of rotational assessment, the inter-observer reproducibility of RBR was not assessed in our study.

## 5. Conclusions

Previous studies highlighted the usefulness of torsional movements and left ventricular twists in the diagnostic process of CTRCD [18,23]. The RBR pattern is simple and more reliable than other proposed torsional parameters. It improves the diagnostic accuracy of GLS. Larger studies are required to correctly define the true clinical value of the RBR pattern.

## Figures and Tables

**Table 1:** Baseline characteristics of the total population and in groups subdivided according to the cardiotoxicity.

	Total Population (*n* = 175)	No CTX (*n* = 153)	CTX (*n* = 22)	*p*
**Age (years)**	55 ± 12	55 ± 12	56 ± 12	ns
**BMI (kg/m^2^)**	24 ± 4	25 ± 4	25 ± 4	ns
**SPB (mmHg)**	125 ± 20	125 ± 13	123 ± 33	ns
**DPB (mmHg)**	80 ± 13	80 ± 14	78 ± 19	ns
**HsTn I (ng/mL)**	0.9 ± 0.6	0.8 ± 0.7	1.7 ± 0.8	ns
**GFR (mL/min)**	99 ± 32	101 ± 34	93 ± 35	ns
**Female gender**	164 (94%)	144 (94%)	20 (91%)	ns
**Hypertension**	48 (27%)	40 (26%)	8 (36%)	ns
**DM**	13 (7%)	11 (7%)	2 (10%)	ns
**Dyslipidaemia**	30 (17%)	26 (17%)	4 (18%)	ns
**Smoking**	28 (16%)	23 (15%)	5 (23%)	ns
**Family history of CV disease**	24 (14%)	19 (13%)	5 (23%)	ns
**CKD**	24 (14)	29 (19%)	5 (23%)	ns
**ACEI/ARBs**	15 (8%)	14 (9%)	1 (5%)	ns
**ARBs**	45 (23%)	40 (26%)	5 (25%)	ns
**BB**	40 (26%)	35 (23%)	5 (25%)	ns
**MRA**	10 (6%)	9 (6%)	1 (5%)	ns
**Diuretics**	22 (13%)	19(13%)	3 (15%)	ns
**CCB**	29 (17%)	26 (17%)	3 (15%)	ns
**ASA**	7 (4%)	6 (4%)	1 (5%)	ns
**OAC**	16 (9%)	14 (9%)	2 (10%)	ns
**Statins**	19 (11%)	17 (11%)	2(10%)	ns

Baseline characteristics of the total population and in groups subdivided according to the cardiotoxicity. Continuous variables are expressed as mean ± standard deviation. ACEI: angiotensin-converting enzyme inhibitor; ARBs: angiotensin receptor blockers; ASA: acetylsalicylic acid; BB: beta-blockers; BMI: body mass index; CCB: calcium channel blockers; CKD: chronic renal disease; CTX: cardiotoxicity; DBP: diastolic blood pressure; DM: diabetes mellitus; GFR: glomerular filtration rate; hs-cTnI: high-sensitivity cardiac troponin I; MRA: mineralocorticoid receptor antagonists; ns; not significant; SPB: systolic blood pressure; OCA: oral anticoagulation.

**Table 2:** Echocardiographic parameters at the basal examination.

	Total Population (*n* = 175)	No CTX (*n* = 153)	CTX (*n* = 22)	*p*
**LVEF (%)**	59.1 ± 10.5	59.0 ± 9.6	54.2 ± 15.9	ns
**LVEDV (mL)**	86.5 ± 19.0	84.1 ± 18.2	95.4 ± 21.3	ns
**LVESV (mL)**	36.4 ± 13.0	34.8 ± 11.9	44.4 ± 18.9	ns
**LAA (cm^2^)**	13.7 ± 8.0	13.2 ± 7.9	16.0 ± 7.4	ns
**RAA (cm^2^)**	11.7 ± 6.5	11.3 ± 6.4	13.1 ± 6.4	ns
**E/A**	1.2 ± 0.9	1.2 ± 1.0	0.9 ± 0.4	ns
**E/E′**	8.1 ± 2.4	7.9 ± 2.3	8.5 ± 3.1	ns
**TAPSE (mm)**	22.3 ± 4.5	22.1 ± 4.2	21.7 ± 6.0	ns
**sPAP (mmHg)**	20.4 ± 12.3	20.6 ± 11.6	16.8 ± 15.2	ns
**GLS (%)**	−18.7 ± 2.7	−18.8 ± 2.4	−16.6 ± 4.6	ns
**LV basal systolic rotation (°)**	−6.3 ± 3.0	−6.3 ± 3.7	−6.1 ± 4.6	ns
**LV basal diastolic rotation (°)**	−2.5 ± 4.5	−2.5 ± 4.7	−2.5 ± 2.3	ns
**LV apical systolic rotation (°)**	7.9 ± 4.9	7.7 ± 4.6	8.3 ± 6.1	ns
**LV apical diastolic rotation (°)**	2.7 ± 3.8	2.6 ± 3.5	3.1 ± 5.5	ns
**LV twisting (°)**	14.4 ± 6.2	14.4 ± 5.6	13.1 ± 10.0	ns
**LV untwisting (°)**	5.4 ± 3.9	5.4 ± 3.6	5.3 ± 5.1	ns

Echocardiographic parameters at the basal examination. Continuous variables are expressed as mean ± standard deviation. GLS: global longitudinal strain; LAA: left atrial area; LV: left ventricle; LVEDV: left ventricular end-diastolic volume; LVEF: left ventricular ejection fraction; LVESV: left ventricular end-systolic volume; ns: not significant; RAA: right atrial area; sPAP: systolic pulmonary arterial pressure; TAPSE: tricuspid anteroposterior systolic excursion.

**Table 3:** GLS and rotation mechanics (RBR) diagnostic power in predicting anthracycline and HER2-induced CTRCD.

Variable	OR	IC 95%	*p*	SE	SP	PPV	PNV
GLS at 3 months (each 1%)	1.84	1.29–2.65	<0.001				
GLS at 6 months (each 1%)	2.01	1.33–3.28	0.001				
GLS ≥ −16% at 3 months	25.63	6.78–95.33	<0.001	73%	90%	76%	89%
GLS ≥ −16% at 6 months	32.51	8.40–126.84	<0.001	77%	91%	77%	91%
RBR at 3 months	9.50	2.18–34.22	0.008	28%	98%	86%	76%
RBR at 6 months	7.00	1.83–26.71	0.004	36%	92%	67%	78%
GLS ≥ −16% or RBR at 3 months	32.64	8.40–126.84	<0.001	77%	91%	77%	91%
GLS ≥ −16% or RBR at 6 months	49.61	11.24–218.99	<0.001	86%	89%	76%	94%

OR: odds ratio, SE: sensitivity, SP: specificity, PPV: predictive positive value; PNV: predictive negative value.

## Data Availability

The data presented in this study is available on request from the corresponding author (the data is not publicly available due to privacy or ethical restrictions).
